# Designing multi‐arm multi‐stage clinical trials using a risk–benefit criterion for treatment selection

**DOI:** 10.1002/sim.6760

**Published:** 2015-10-12

**Authors:** Thomas Jaki, Lisa V. Hampson

**Affiliations:** ^1^ Medical and Pharmaceutical Statistics Research Unit, Department of Mathematics and StatisticsLancaster UniversityLancasterU.K.

**Keywords:** familywise error rate, multi‐arm multi‐stage (MAMS), multiple endpoints, safety, treatment selection

## Abstract

Multi‐arm clinical trials that compare several active treatments to a common control have been proposed as an efficient means of making an informed decision about which of several treatments should be evaluated further in a confirmatory study. Additional efficiency is gained by incorporating interim analyses and, in particular, seamless Phase II/III designs have been the focus of recent research. Common to much of this work is the constraint that selection and formal testing should be based on a single efficacy endpoint, despite the fact that in practice, safety considerations will often play a central role in determining selection decisions. Here, we develop a multi‐arm multi‐stage design for a trial with an efficacy and safety endpoint. The safety endpoint is explicitly considered in the formulation of the problem, selection of experimental arm and hypothesis testing. The design extends group‐sequential ideas and considers the scenario where a minimal safety requirement is to be fulfilled and the treatment yielding the best combined safety and efficacy trade‐off satisfying this constraint is selected for further testing. The treatment with the best trade‐off is selected at the first interim analysis, while the whole trial is allowed to compose of *J* analyses. We show that the design controls the familywise error rate in the strong sense and illustrate the method through an example and simulation. We find that the design is robust to misspecification of the correlation between the endpoints and requires similar numbers of subjects to a trial based on efficacy alone for moderately correlated endpoints. © 2015 The Authors. Statistics in Medicine Published by John Wiley & Sons Ltd.

## 1 Introduction

Prior to undertaking a confirmatory Phase III clinical trial, there is often uncertainty about which treatment should be selected for evaluation from a number of candidates. Here, treatments could be different doses of the same drug or different combinations of multiple drugs. Uncertainty about which treatment to select often stems from the fact that early phase trials typically evaluate medicines in different populations, using different endpoints, to those that will be the focus of confirmatory studies. The current high failure rate of Phase III trials of around 50% 1 combined with their substantial cost 2 make selecting an appropriate treatment for evaluation in Phase III of paramount importance.

As an efficient solution to this problem, designs for seamless Phase II/III multi‐arm clinical trials have been proposed, which compare several active treatments with a common control group. Phase II of the study is used to learn about all treatments. At the end of this first stage, one or more of the active treatments is selected and taken forward with control for evaluation in Phase III. Data accumulated across both stages of the trial are used to test whether the selected treatment(s) is(are) superior to control at the end of the study. The simultaneous comparison of several treatments means that expected sample sizes and durations of multi‐arm trials are markedly smaller than the alternative of evaluating each treatment separately. For added efficiency, solutions that incorporate a series of interim analyses to allow early stopping either for efficacy or to drop ineffective treatments have recently received attention 3, 4, 5, 6, 7. The approaches discussed in the literature to date can be characterized by two main differences. The first is the underlying statistical framework that either generalizes group sequential designs 8, 9 to accommodate multiple treatment arms 3 or makes use of *p*‐value combination rules within closed testing procedures 10. The second difference is the way in which treatments are selected. In 3, for example, only the best performing treatment is selected at the first interim analysis and subsequently compared with control over multiple stages, while in 6, all treatments surpassing a threshold at each stage are continued. Meanwhile, Kelly *et al.*, 11 advocate a rule that selects all treatments close to the best performing treatment at the first interim analysis.

A further commonality of several of the approaches discussed in the literature is the assumption of normally distributed data and the fact that a single endpoint is considered. However, there are exceptions. For results for non‐normal endpoints, see, for example, 12, 13, 14. More generally, adaptive procedures using *p*‐value combination rules within closed testing procedures make no assumptions about the distribution of patient responses nor place any constraints on the form of the treatment selection rule: the only constraint is that *p*‐values for testing elementary and intersection null hypotheses must follow a Uniform(0,1) (or stochastically larger) distribution under the null 5. For procedures that consider more than endpoint, see 15, which describes a seamless Phase II/III trial using a composite rule based on two hierarchically ordered efficacy endpoints to guide treatment selection decisions, as well as relevant safety data; to adjust for multiple testing, pairwise comparisons of selected treatments against placebo are adjusted using a Bonferroni correction. Early phase oncology trials also often assess efficacy and monitor toxicity, see 16 for a Phase I/II trial design combining time‐to‐response and time‐to‐toxicity endpoints into a single statistic used for interim decision making, weighting pairs of outcomes according to utilities elicited from experts. In other areas, such as mental health, co‐primary efficacy endpoints are measured, and no single measure is accepted as definitive. Although it is sometimes sensible to combine different endpoints into a single test statistic, substantial gains in efficiency can be achieved if they are evaluated jointly, especially when endpoints capture the effects of a treatment on different aspects of the disease. Furthermore, combining information obtained on efficacy and safety endpoints into a single test statistic will be inappropriate because good efficacy will not compensate for poor safety in practice.

Methods for two‐arm group‐sequential trials with multivariate normal endpoints 17, 18, two binary endpoints 19 and a mixture of time‐to‐event and nonfailure endpoints 20 have been developed. In this article, we develop a multi‐arm multi‐stage (MAMS) design for a trial with an efficacy endpoint and a safety endpoint. The novelty of the proposed design is that it is based on a joint model for the efficacy and safety outcomes, while information on both endpoints is incorporated into treatment selection decisions. We consider the situation where a minimal safety requirement is to be fulfilled and the treatment with the best combined safety and efficacy trade‐off satisfying this constraint is selected for further testing. Selection is made at the first interim analysis, while the whole trial is allowed to compose of *J* analyses. Final decisions about a selected treatment are based on tests of efficacy and safety relative to control. In Section 2, we show that the design controls the familywise error rate (FWER) in the strong sense and discuss methods for sample size calculations. In Section 3, we illustrate the method through an example and simulations based on the Telmisartan and Insulin Resistance in HIV (TAILoR) study, a multi‐arm trial of treatments to reduce insulin resistance in human immunodeficiency virus‐positive patients. We conclude in Section 4 with a discussion of our findings and avenues for future research.

## 2 Statistical framework

We propose MAMS designs that begin in Stage 1 by comparing *K* active treatments with a common control group. The overall objective of the trial is to select the ‘best’ of the *K* treatments and then make comparisons with the control. Rather than be based solely on efficacy, treatment selection decisions will often reflect a compromise between the potential benefits and side effects of a new therapy. For example, a new treatment may need to demonstrate non‐inferior safety and superior efficacy to represent a clinically meaningful advantage over a well‐understood control. We propose designs that explicitly account for the impact of safety considerations on decision‐making. Throughout, we restrict attention to the case where a single treatment is selected at the first analysis. We begin by focusing attention on a single‐stage design and discuss the natural extension to multiple stages in Section 2.4.

### 2.1 Treatment selection rules

Suppose the trial proceeds in Stage 1 by measuring a bivariate endpoint on each patient. Labelling control as treatment 0, let *Y*_*Eik*_ and *Y*_*Sik*_ represent the efficacy and safety responses, respectively, of subject *i* on treatment *k*, which can be modelled as

$$\left(\begin{array}{c} Y_{\mathrm{Eik}}\\ Y_{\mathrm{Sik}} \end{array}\right)∼N\left(\left(\begin{array}{c} \mu_{\mathrm{Ek}}\\ \mu_{\mathrm{Sk}} \end{array}\right),\left(\begin{array}{cc} \sigma_{E}^{2} & \rho \sigma_{E}\sigma_{S}\\ \rho \sigma_{E}\sigma_{S} & \sigma_{S}^{2} \end{array}\right)\right),\;i=1,\ldots ,n;k=0,1,\ldots ,K,$$

where *ρ* is the within‐subject correlation, and we assume that the variance–covariance matrix of responses is known. Let *θ*_*Ek*_=*μ*_*Ek*_−*μ*_*E*0_ and *θ*_*Sk*_=*μ*_*Sk*_−*μ*_*S*0_ measure the advantage of treatment *k* over control for efficacy and safety, respectively, where we will assume that increases in response are desirable for both endpoints. Thus, ***θ*** = (***θ***_*E*_,***θ***_***S***_) is a vector of length 2*K* containing the efficacy and safety effects of the *K* treatments. For each treatment *k* = 1,…,*K*, we define two hypotheses *H*_*Ek*_:*θ*_*Ek*_≤0 and *H*_*Sk*_:*θ*_*Sk*_≤0. The null hypothesis we wish to test is *H*_0*k*_:*H*_*Ek*_∪*H*_*Sk*_ stating that treatment *k* is either ineffective or unsafe in comparison with control; rejecting *H*_0*k*_ implies that treatment *k* is both effective and safe. The global null hypothesis $H_{0}:\;\overset{K}{\underset{k=1}{⋂}}H_{0k}$ represents the case that all *K* treatments are either unsafe or ineffective. For ease of presentation, we consider tests of superiority, although Jennison and Turnbull 18 observe that it is straightforward to accommodate tests of non‐inferiority in this framework by subtracting the non‐inferiority margin (for a difference in means) from patient responses on the control treatment.

For presentational purposes, we assume a common 1:1 allocation of patients to each of the *K* active treatments and control and denote the number of patient responses available on each arm by *n*. Thus, at the end of Stage 1, for each *k* = 1,…,*K*, Fisher's information for *θ*_*Tk*_ takes a common value denoted by $I_{T}=n/(2\sigma_{T}^{2})$, for *T*∈*E*,*S*. In Appendix A.1 of the Supporting Information, we outline how the procedure could be extended to accommodate a common *r*:1 allocation of patients to active treatments and control. Define ${\hat{\mu }}_{\mathrm{Tk}}$ as the maximum likelihood estimator of *μ*_*Tk*_. Accumulated data on each treatment are summarized by the bivariate score statistic

(1) $$\left(\begin{array}{c} Z_{\mathrm{Ek}}\\ Z_{\mathrm{Sk}} \end{array}\right)=\left(\begin{array}{c} I_{E}\left({\hat{\mu }}_{\mathrm{Ek}}-{\hat{\mu }}_{E0}\right)\\ I_{S}\left({\hat{\mu }}_{\mathrm{Sk}}-{\hat{\mu }}_{S0}\right) \end{array}\right)∼N\left(\left(\begin{array}{c} I_{E}\theta_{\mathrm{Ek}}\\ I_{S}\theta_{\mathrm{Sk}} \end{array}\right),\left(\begin{array}{cc} I_{E} & \rho \sqrt{\left(I_{E}I_{S}\right)}\\ \rho \sqrt{\left(I_{E}I_{S}\right)} & I_{S} \end{array}\right)\right).$$

Only treatments meeting a pre‐specified minimum safety requirement may be considered for selection. Let $N_{S}$ denote the number of treatments eligible for selection, which are indexed by the selection set $SS=\{k:\;Z_{\mathrm{Sk}}>c\}$. If $N_{S}=0$, the test is stopped for futility without rejecting *H*_0_. Otherwise, we select from SS the treatment maximizing the objective function

(2) $$O_{k}=\frac{w_{E}Z_{\mathrm{Ek}}}{\sqrt{I_{E}}}+\frac{w_{S}Z_{\mathrm{Sk}}}{\sqrt{I_{S}}},$$

where *w*_*E*_ and *w*_***S***_ are pre‐specified non‐negative weights satisfying $w_{E}^{2}+w_{S}^{2}=1$. Unplanned deviations from the pre‐specified treatment selection rule could lead to inflation of the FWER above the nominal level. One of the motivations of this design is, however, to formally include safety in the decision‐making so that such deviations become less frequent. Should unexpected modification be necessary, however, conditional error principle 21 can be used to maintain FWER control. It is worth pointing out that we incorporate the safety threshold because the objective function allows good efficacy to compensate for poor safety. In practice, this would only be acceptable up to a certain point, which is defined by the safety threshold. A natural choice for this threshold in our opinion is *c* = 0, that is, we only select from treatments with comparable or better safety than control in stage 1, although in principle other values could be used instead.

Let *i* ^⋆^ index the treatment selected from Stage 1 on the basis of the objective function $O_{i^{⋆}}=\max_{k\in SS}\{O_{k}\}$. Because the selected treatment will only be declared preferable to control if we can reject the null hypothesis $H_{0i^{⋆}}:H_{Ei^{⋆}}\cup H_{Si^{⋆}}$, a natural choice of weights is $w_{E}=w_{S}=\sqrt{0.5}$ as this ensures consistency between selection decisions and the final analysis of the trial.

We propose single‐stage tests of *H*_01_,…,*H*_0*K*_ with stopping rules of the form:

(3) $$\begin{array}{cc} \text{If}Z_{S1},\ldots ,Z_{\mathrm{SK}}\leq c & \text{Stop and accept}H_{0}\\ \text{Otherwise} & \text{Select from}SS\text{treatment}i^{⋆}\text{maximizing objective function}O\\ \text{and conduct the final analysis.}\\ \text{At the final analysis:}\\ \text{If}Z_{Ei^{⋆}}\geq u_{E}\text{and}Z_{Si^{⋆}}\geq u_{S} & \text{Stop and reject}H_{0}\text{in}\mathrm{favourof}H_{1i^{⋆}}:\{\theta_{Ei^{⋆}}>0\}\cap \{\theta_{Si^{⋆}}>0\},\\ \text{Otherwise} & \text{Stop and accept}H_{0}. \end{array}$$

At the final analysis of the proposed test, superiority can only be claimed for the selected treatment *i* ^⋆^. Consequently, we define the FWER of the procedure as $P\{\text{Reject}H_{0}\text{in favour of a false}H_{1i^{⋆}};\;\theta \}$. This probability depends on both the minimum safety requirement, *c*, and the stopping boundaries (*u*_*E*_,*u*_***S***_). Our approach is to fix *c* = 0 and find the pair of critical values maintaining strong control of the FWER at level *α*. This criterion stipulates that $P\{\text{Reject}H_{0}\text{in favour of a false}H_{1i^{⋆}};\;\theta \}\leq \alpha$ for all configurations of ***θ*** with at least one *θ*_*Tk*_≤0, for *T*∈{*E*,*S*} and k∈{1,…,K}. If $H_{01},\ldots ,H_{0K}$ are all true, a familywise error is made if the test terminates with rejection of $H_{0i^{⋆}}$ whatever treatment is selected, and the FWER is given by $P\{Z_{Ei^{⋆}}\geq u_{E},Z_{Si^{⋆}}\geq u_{S},N_{S}\geq 1;\;\theta \}$. In the remainder of this section, we discuss how to find (*u*_***S***_,*u*_*E*_) maintaining strong control of the FWER.

### 2.2 Specification of test boundaries

We propose choosing the boundaries of test (3) to ensure the FWER is controlled at level *α* as we approach the following two ‘worst‐case’ limiting configurations of *θ*: (1) ***θ***_*E*_=(*∞*,…,*∞*), ***θ***_***S***_=(0,…,0) and (2) ***θ***_*E*_=(0,…,0), ***θ***_***S***_=(*∞*,…,*∞*). We claim that specifying test boundaries according to this criterion ensures strong control of the FWER and prove this claim using a combination of analytical arguments and simulation. This result agrees with the findings of 18 for the case that *K* = 1. Let *Γ* represent a set indexing treatments with positive efficacy and safety effects. We begin considering a subset of the null parameter space comprising configurations of ***θ*** such that

- For all treatments *k* with *k* ∉ *Γ*, *θ*_*Ek*_≤0 and *θ*_*Sk*_≤0; or
- For all treatments *k* with *k* ∉ *Γ*, $\theta_{\mathrm{Ek}}\geq 0$ and *θ*_*Sk*_≤0; or
- For all treatments *k* with *k* ∉ *Γ*, *θ*_*Ek*_≤0 and $\theta_{\mathrm{Sk}}\geq 0$.

Under the global null hypothesis, *Γ* = *∅*, and the constraints on ***θ*** configurations defined previously correspond to assuming that effects of different treatments on the same endpoint have the same sign. We claim that the FWER of procedure (3) under configurations of ***θ*** in this restricted global null parameter space is maximized under constellations with ***θ***_*E*_=(*γ*_*E*_,…,*γ*_*E*_) and ***θ***_***S***_=(*γ*_***S***_,…,*γ*_***S***_), and furthermore that local maxima of the FWER are attained in the limit as *γ*_***S***_→*∞* and *γ*_*E*_=0, and in the limit as *γ*_*E*_→*∞* and *γ*_***S***_=0.

To prove these claims, we begin by assuming that all treatments are always eligible for selection and consider the configuration of ***θ*** with ***θ***_*E*_=(*γ*_*E*_,…,*γ*_*E*_) and ***θ***_***S***_=(*γ*_***S***_,…,*γ*_***S***_). Then, letting some elements of ***θ***_***S***_ fall below *γ*_***S***_ decrease stochastically the distribution of $\left(Z_{Ei^{⋆}},Z_{Si^{⋆}}\right)$ as both statistics tend to take lower values on average. To explain this, note that since all treatments remain competitive for efficacy, treatments must perform well for *Z*_***S***_ if they are to rank highly for the objective function *O*. Thus, selection decisions are, in effect, driven primarily by safety data so that a treatment may beat its competitors on the basis of *O* with lower values of *Z*_*E*_. Letting some of the *θ*_*Sk*_ *s* drop below *γ*_***S***_ also decreases stochastically the distribution of $Z_{Si^{⋆}}$ too: the treatment associated with the largest element of ***θ***_***S***_ is now ‘safest’ by some margin, meaning that on average, lower values of *Z*_***S***_ will be sufficient for it to beat the weaker competition to ensure selection. Similar arguments imply keeping ***θ***_***S***_ fixed at ***γ***_***S***_ and letting some elements of ***θ***_*E*_ fall below *γ*_*E*_ decreases stochastically the distributions of $Z_{Ei^{⋆}}$ and $Z_{Si^{⋆}}$. On the other hand, simultaneously forcing elements of ***θ***_*E*_ below *γ*_*E*_ and elements of ***θ***_***S***_ below *γ*_***S***_ decreases stochastically the distribution of $\left(Z_{Ei^{⋆}},Z_{Si^{⋆}}\right)$: systematic differences between treatments imply that it is possible for a good safety profile to compensate for poor efficacy (and vice versa) resulting in lower average values of *Z*_*E*_ and *Z*_***S***_ for the selected treatment.

Letting ***θ***_*E*_=(*γ*_*E*_,…,*γ*_*E*_) and ***θ***_***S***_=(*γ*_***S***_,…,*γ*_***S***_), increasing *γ*_*E*_ or *γ*_***S***_ increases the probability of rejecting *H*_0_. Thus, looking across the restricted global null parameter space, the probability of making a familywise error is maximized at the boundaries of the space, that is, in the limit as *γ*_***S***_→*∞* and *γ*_*E*_=0, and in the limit as *γ*_*E*_→*∞* and *γ*_***S***_=0. If for some treatment *k*, *θ*_*Ek*_ and *θ*_*Sk*_ are both positive so that *Γ* ≠ *∅*, this treatment will be more likely to be selected, in which case we cannot commit a familywise error and the FWER decreases. Therefore, controlling the FWER for all configurations of ***θ*** in the restricted global null parameter space ensures the FWER is controlled over the wider null parameter space defined previously.

So far, we have consider the case that all treatments are always eligible for selection, in effect setting *c* =− *∞*. However, we claim that for general values of *c*, local maxima of the FWER are attained in the limit as we approach the worst case configurations of ***θ*** identified previously. This is because under this requirement, the expected size of SS is determined by *γ*_***S***_. Therefore, increasing *γ*_***S***_ increases stochastically the distribution of $\left(Z_{Ei^{⋆}},Z_{Si^{⋆}}\right)$ as the average number of treatments from which we can select increases. In particular, as *γ*_***S***_ approaches *∞*, SS includes all *K* treatments almost surely and rejects *H*_0_ if $Z_{Ei^{⋆}}>u_{E}$. The probability of falsely rejecting *H*_0_ is then maximized for *γ*_*E*_=0. Similarly, setting *γ*_***S***_=0, the FWER reaches a second local maximum as *γ*_*E*_→*∞*. To see this note that for *γ*_***S***_=0, inclusion of treatments in the selection set is random so that the probability of rejection is maximized for maximal effect on efficacy.

To complete our justification for designing test (3) to control the FWER under the ‘worst‐case’ limiting configurations of ***θ***, we go beyond the arguments stated previously to claim that this approach ensures strong control of the FWER. In particular, tests defined in this way will control the FWER for any configuration of ***θ*** with ***θ***_*E*_=(*θ*_*E*1_,…,*θ*_*EK*_) and ***θ***_***S***_=(*θ*_*S*1_,…,*θ*_*SK*_), where one *θ*_·*i*_ is zero and the other *∞*. While we cannot prove these claims analytically, we evaluate them via simulation in Section 3.2. Assuming for now that these claims do hold, it is appropriate to choose boundaries (*u*_*E*_,*u*_***S***_) to ensure that

(4) $$\underset{\gamma_{S}\to \infty }{\lim}P\{Z_{Ei^{⋆}}\geq u_{E},Z_{Si^{⋆}}\geq u_{S}|\;N_{S}\geq 1;\theta_{E}=(0,\ldots ,0),\theta_{S}=(\gamma_{S},\ldots ,\gamma_{S})\}=\alpha ,$$

(5) $$\underset{\gamma_{E}\to \infty }{\lim}P\{Z_{Ei^{⋆}}\geq u_{E},Z_{Si^{⋆}}\geq u_{S}|\;N_{S}\geq 1;\theta_{E}=(\gamma_{E},\ldots ,\gamma_{E}),\theta_{S}=0\}=\alpha /P\{N_{S}\geq 1;\theta_{S}=0\},$$

because $\underset{\gamma_{S}\to \infty }{\lim}P\{N_{S}\geq 1;\theta_{S}=(\gamma_{S},\ldots ,\gamma_{S})\}=1$ and the probability that at least one treatment meets the minimum safety criterion does not depend on ***θ***_*E*_. As *γ*_***S***_ and *γ*_*E*_ approach *∞*, the bivariate probabilities on the left hand sides (LHSs) of (4) and (5) converge to univariate probabilities. We find (*u*_*E*_,*u*_***S***_) so that the limits of these marginal rejection probabilities are equal to the values required to ensure FWER control. Limits of rejection probabilities are found by integrating the limits of the marginal conditional densities of $Z_{Ei^{⋆}}$ and $Z_{Si^{⋆}}$ derived in Appendix A.2 of the Supporting Information. It is important to note that these marginal densities depend on the correlation coefficient *ρ*. So far, we have assumed that this parameter is known. In Section 3.3, we explore the robustness of attained FWERs to misspecification of *ρ*. Marginal densities of test statistics depend on variances only through the information levels $I_{E}$ and $I_{S}$. The effect of assuming a known variance has previously been investigated in similar settings 22, and the quantile substitution approach described in 8 has been shown to work well.

### 2.3 Sample size calculations

We wish to calculate the sample size needed for test (3) to attain a disjunctive power, that is, probability of rejecting at least one false null hypothesis 5, 23, of 1 − *β* under the configuration of ***θ*** with ***θ***_*E*_=(*δ*_0_,…,*δ*_0_,*δ*) and ***θ***_***S***_=(*γ*_***S***_,…,*γ*_***S***_) with *γ*_***S***_>0. We may approximate further by letting *γ*_***S***_→*∞*, which can be justified by the belief that a potentially unsafe treatment is unlikely to be included in the trial. In this case, a test's power simplifies to

(6) $$\begin{array}{c} \underset{\gamma_{S}\to \infty }{\lim}P\{Z_{Ei^{⋆}}\geq u_{E},Z_{Si^{⋆}}\geq u_{S}|\;N_{S}\geq 1;\theta_{E}=(\delta_{0},\ldots ,\delta_{0},\delta ),\theta_{S}=(\gamma_{S},\ldots ,\gamma_{S})\}\\ \;=\underset{\gamma_{S}\to \infty }{\lim}P\{Z_{Ei^{⋆}}\geq u_{E}|\;N_{S}\geq 1;\theta_{E}=(\delta_{0},\ldots ,\delta_{0},\delta ),\theta_{S}=(\gamma_{S},\ldots ,\gamma_{S})\}, \end{array}$$

and limiting probabilities are found by integrating the limits of the marginal conditional density of $Z_{Ei^{⋆}}$. Using the results of Appendix A.1 and following the workings of Appendix A.2.1 of the Supporting Information, we can show that limiting rejection probability (6) is given by

(7) $$\begin{array}{c} (K-1)\overset{\infty }{\underset{-\infty }{\int }}\overset{\infty }{\underset{-\infty }{\int }}\overset{\infty }{\underset{-\infty }{\int }}f_{X_{E0}|X_{S0}}\left(x_{1}-\delta_{0}I_{E}|y\right)\;f_{X_{S0}}(y)\;f_{X_{S0}}(y-m)P{\left\{U\leq \ell_{4}\right\}}^{K-2}P\left\{U\leq \ell_{5}\right\}\\ \;\;\;\;\;\times \Phi \left(\frac{x_{1}-\sqrt{I_{E}/I_{S}}\rho (y-m)-u_{E}}{\sqrt{\left(I_{E}/2)(1-\rho^{2}\right)}}\right)dx_{1}\;dy\;dm\\ +\overset{\infty }{\underset{-\infty }{\int }}\overset{\infty }{\underset{-\infty }{\int }}\overset{\infty }{\underset{-\infty }{\int }}f_{X_{E0}|X_{S0}}\left(x_{1}-\delta I_{E}|y\right)\;f_{X_{S0}}(y)\;f_{X_{S0}}(y-m)P{\left\{U\leq \ell_{4}\right\}}^{K-1}\\ \;\;\;\;\;\times \Phi \left(\frac{x_{1}-\sqrt{I_{E}/I_{S}}\rho (y-m)-u_{E}}{\sqrt{\left(I_{E}/2\right)\left(1-\rho^{2}\right)}}\right)dx_{1}\;dy\;dm, \end{array}$$

where *U* ∼ *N*(0,*w*_*E*_ *w*_***S***_ *ρ* + 0.5), $\sqrt{\left(I_{E}I_{S}\right)}\ell_{4}=\sqrt{I_{E}}w_{S}y+\sqrt{I_{S}}w_{E}(x_{1}-I_{E}\delta_{0})$ and $\sqrt{\left(I_{E}I_{S}\right)}\ell_{5}=\sqrt{I_{E}}w_{S}y+\sqrt{I_{S}}w_{E}(x_{1}-I_{E}\delta )$. For computational convenience, we proceed assuming that $I_{E}=I_{S}=I_{1}$ and conduct a one‐dimensional search to find the common information level $I_{1}^{⋆}$ for which rejection probability (7) equals 1 − *β*; at each iteration of this search, boundaries for monitoring score statistics $Z_{Ei^{⋆}}$ and $Z_{Si^{⋆}}$ are updated to ensure strong control of the FWER at level *α* under the proposed information level. Because information level $I_{1}^{⋆}$ typically corresponds to requiring fractions of subjects, in practice, we propose rounding up the total sample size to $n^{⋆}=2\;\max\{\sigma_{E}^{2}I_{1}^{⋆},\;\sigma_{S}^{2}I_{1}^{⋆}\}$ patients per treatment arm. The test is then applied with critical values calculated for information levels $I_{E}=n^{⋆}/(2\sigma_{E}^{2})$ and $I_{S}=n^{⋆}/(2\sigma_{S}^{2})$. If a procedure's power is monotone increasing in $I_{E}$ and $I_{S}$, this sample size criterion will be conservative in the sense that attained power will exceed 1 − *β*. In Section 3.2, we use simulation to evaluate properties of tests designed according to the proposed sample size criterion.

### 2.4 Beyond single‐stage designs

It is straightforward to extend our approach to find designs maintaining control of the FWER when multiple interim analyses are planned. Let *Z*_*Tk*,*j*_ denote the score statistic at interim analysis j for endpoint T on treatment k. A multi‐stage test of *H*_01_,…,*H*_0*K*_ has a stopping rule of the form:

(8) $$\begin{array}{cc} \text{At the end of stage 1:}\\ \text{If}Z_{S1,1},\ldots ,Z_{\mathrm{SK},1}\leq c & \text{Stop and accept}H_{0}\\ \text{Otherwise} & \text{Select from}SS\text{treatment}i^{⋆}\text{maximizing objective function}O\\ \text{and conduct interim analysis 1.}\\ \text{At interim analysis}j=1,\ldots ,J:\\ \text{If}Z_{Ei^{⋆},j}\geq u_{\mathrm{Ej}}\text{and}Z_{Si^{⋆}}\geq u_{\mathrm{Sj}} & \text{Stop and reject}H_{0}\text{in favour of}H_{1i^{⋆}}:\{\theta_{Ei^{⋆}}>0\}\cap \{\theta_{Si^{⋆}}>0\},\\ \text{If}Z_{Ei^{⋆},j}\leq l_{\mathrm{Ej}}\text{or}Z_{Si^{⋆},j}\leq l_{\mathrm{Sj}} & \text{Stop and accept}H_{0},\\ \text{Otherwise} & \text{Continue to interim analysis}j+1. \end{array}$$

Multi‐stage tests are defined with binding futility rules so that if either $Z_{Ei^{⋆},j}\leq l_{\mathrm{Ej}}$ or $Z_{Si^{⋆},j}\leq l_{\mathrm{Sj}}$, the procedure must stop immediately at interim analysis *j* without declaring treatment *i* ^⋆^ safe and effective. Criteria (4) and (5) imply that we can uncouple the searches needed to find critical values for monitoring efficacy and safety score statistics. Furthermore, for *T*∈{*E*,*S*}, increments $Z_{Ti^{⋆},2}-Z_{Ti^{⋆},1},\ldots ,Z_{Ti^{⋆},J}-Z_{Ti^{⋆},J-1}$ are independent and follow the same distribution as increments in score statistics generated by a univariate group sequential test (GST) without selection 3. Thus, we can find (*l*_*E*1_,*u*_*E*1_),…,(*l*_*EJ*_,*u*_*EJ*_) as the boundaries defining a one‐sided univariate GST monitoring $\left\{Z_{Ei^{⋆},1},\ldots ,Z_{Ei^{⋆},J}\right\}$ with limiting conditional type I error rate *α* given $N_{S}\geq 1$ under *γ*_*E*_=0 and letting *g* *a* *m* *m* *a*_***S***_→*∞*. Following 3, we propose that an alpha‐spending approach 24 be used to find the upper and lower boundaries at each stage *j* = 1,…,*J* satisfying

$$\begin{array}{cc} \underset{\gamma_{S}\to \infty }{\lim}P & \left\{Z_{Ei^{⋆},1}\in \left(l_{E1},\;u_{E1}\right),\ldots ,Z_{Ei^{⋆},j-1}\right.\in \left(l_{E(j-1)},u_{E(j-1)}\right),Z_{Ei^{⋆},j}\geq u_{\mathrm{Ej}}|\;N_{S}\geq 1;\theta_{E}=0,\theta_{S}\\ =\left.\left(\gamma_{S},\ldots ,\gamma_{S}\right)\right\}=f_{U}\left(t_{j}\right)-f_{U}\left(t_{j-1}\right)\\ \underset{\gamma_{S}\to \infty }{\lim}P & \left\{Z_{Ei^{⋆},1}\in \left(l_{E1},\;u_{E1}\right),\ldots ,Z_{Ei^{⋆},j-1}\right.\in \left(l_{E(j-1)},u_{E(j-1)}\right),Z_{Ei^{⋆},j}\leq l_{\mathrm{Ej}}|\;N_{S}\geq 1;\theta_{E}\\ =0,\theta_{S}=\left.\left(\gamma_{S},\ldots ,\gamma_{S}\right)\right\}=f_{L}\left(t_{j}\right)-f_{L}\left(t_{j-1}\right), \end{array}$$

where *t*_*j*_ is the fraction of $I_{\mathrm{EJ}}$, the maximum information level for the efficacy treatment effect, accumulated by stage *j*, and *f*_*U*_ and *f*_*L*_ are monotone increasing functions satisfying *f*_*U*_(0) = *f*_*L*_(0) = 0 and, for t≥1, *f*_*U*_(*t*) = *α* and *f*_*L*_(*t*) = 1−*α*. A similar process can be used to find the boundaries (*l*_*S*1_,*u*_*S*1_),…,(*l*_*SJ*_,*u*_*SJ*_) for monitoring $\left\{Z_{Si^{⋆},1},\ldots ,Z_{Si^{⋆},J}\right\}$. Safety boundaries are determined using *f*_*L*_ and *f*_*U*_ to spend error probabilities as a function of the observed information for $\theta_{E,i^{⋆}}$; this ensures that *u*_*EJ*_=*l*_*EJ*_ and *u*_*SJ*_=*l*_*SJ*_, so that procedure (8) terminates properly at analysis *J* with a final hypothesis decision for any choice of $I_{\mathrm{EJ}}$ even when the variances of the efficacy and safety endpoints differ. To find the required sample size, we follow Section 2.3 and search for the maximum information level $I_{\mathrm{EJ}}$ for which the test has power 1 − *β* according to criterion (6) under an anticipated information sequence $I_{E1},\ldots ,I_{\mathrm{EJ}}$, setting each $I_{\mathrm{Sj}}=\sigma_{E}^{2}I_{\mathrm{Ej}}/\sigma_{S}^{2}$ to account for differences between the rates at which information on safety and efficacy effects accumulate. The test will recruit up to $n^{⋆}=2\sigma_{E}^{2}I_{\mathrm{EJ}}$ patients on the selected treatment and control in the absence of early stopping.

## 3 Example

In this section, we will examine the operating characteristics of the proposed designs through a series of examples motivated by the TAILoR study, a MAMS trial comparing several doses of telmisartan with control for the reduction of insulin resistance in human immunodeficiency virus‐positive patients receiving combination antiretroviral therapy 6. The study, which is currently ongoing, uses the change in the Homeostatic model assessment ‐ Insulin resistance (HOMA‐IR) index between baseline and 24weeks as the efficacy endpoint.

In this section, we imagine how the TAILoR study might have been designed as a single‐stage procedure of the form shown in (3), using the methodology described in this paper to incorporate a safety endpoint in addition to the efficacy endpoint used in the ongoing study. A plausible safety endpoint is change in systolic blood pressure from baseline because telmisartan is licensed for the treatment of hypertension. An excessive drop in blood pressure for patients without hypertension would be considered an undesirable safety risk. With the exception of this modification, we will assume the design parameters of the original TAILoR study. We therefore stipulate an FWER of 0.05 and seek designs randomizing patients equally across treatment arms with power 0.9 to correctly reject one false null hypothesis. When the TAILoR study was first designed, four doses of telmisartan were planned. For consistency with previous publications 22, 25, we consider the scenario that *K* = 4 active treatments are to be compared with control, despite the ongoing study using three doses because of last minute changes to the study. The standardized desirable effect for efficacy, *δ*, used for sample size calculations is set as 0.545, and the minimum clinically important difference is defined as 0.178. Under the assumption that all treatments are truly safe, we do not require specification of an effect on safety when using (7) for sample size calculations. However, if such an assumption is undesirable, Equation (6) in Appendix A.1 of the Supporting Information can be used with the anticipated safety effect. Boundary calculations and sample size determinations require us to numerically evaluate multi‐dimensional integrals. For this purpose, we used the R package cubature 26 and verified solutions for the obtained boundaries using 100000‐fold simulations.

### 3.1 Design options

**Figure 1:**
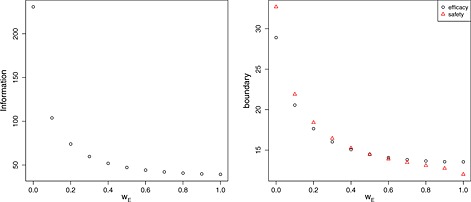
Information per arm, $I_{1}^{⋆}$ (left), and safety/efficacy stopping boundary (right) needed for tests of the form (3) to maintain strong control of the FWER at level 0.05 and attain limiting power 0.9 when ***θ***_*E*_=(0.178,0.178,0.178,0.545) and all treatments are safe. Designs are found for tests making treatment selection decisions according to objective function (2) assuming *σ*_*E*_=*σ*_***S***_=1 and *ρ* = 0.4.

Figure 1 shows how the required information per arm and safety/efficacy stopping boundaries vary as the weight *w*_*E*_ changes. The within‐subject correlation of efficacy and safety responses is assumed to be 0.4. The information required is largest when selection of the treatment is based only on the safety endpoint (*w*_*E*_=0), while it decreases as the weight on efficacy increases. Similarly, both the efficacy and safety boundaries decrease as the required information decreases, as expected. There is, however, an apparent difference between the efficacy and safety boundary, depending on the weight given to each endpoint. For small weights on efficacy, the efficacy boundary is smaller than the safety boundary, while this pattern reverses once more weight is attributed to efficacy for selection. For equal weights, the boundaries for efficacy and safety are identical.

### 3.2 Error rates

**Figure 2:**
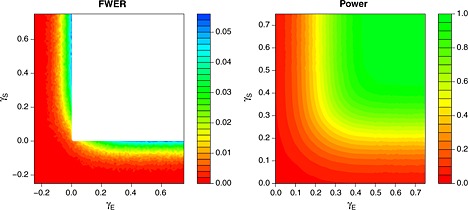
Empirical FWERs over the null space (left) and power to correctly reject at least one false null hypothesis (right) of tests of the form (3) designed to maintain strong control of the FWER at level 0.05 and to attain power 0.9 under ***θ***_*E*_=(0.178,0.178,0.178,0.545) (when all treatments are safe). Tests are designed and conducted with *K* = 4, $w_{E}^{2}=w_{S}^{2}=0.5$, *ρ* = 0.4 and *σ*_*E*_=*σ*_***S***_=1. FWER and power are evaluated under configurations of ***θ*** with ***θ***_***S***_=(*γ*_***S***_,…,*γ*_***S***_) and ***θ***_*E*_=(*γ*_*E*_,…,*γ*_*E*_). Results are based on 10000 simulations for each parameter configuration.

In this section, we illustrate properties of tests of the form (3) designed and conducted with equal weights *w*_*E*_=*w*_***S***_ and correlation coefficient *ρ* = 0.4. Under this setting, the information required per arm is $I_{1}^{⋆}=47.148$(*n* = 94.296), and the stopping boundaries are *u*_*E*_=*u*_***S***_=14.466. Empirical error rates based on 10000 simulation runs for each point on a grid of parameters are shown in Figure 2 for cases where all treatments have the same pair of effects (*γ*_*E*_,*γ*_***S***_) versus control. The left‐hand panel clearly shows that the FWER of the design is maximized if one of the effects is at the boundary of the null space and the other is large. As expected, the power of the design increases as at least one of the effects increases.

**Figure 3:**
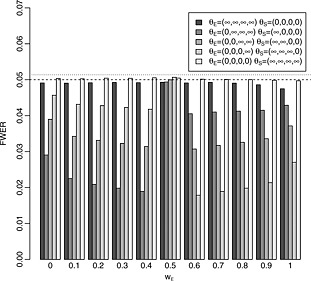
Empirical FWER for different configurations of the true effects for tests of the form (3) designed to maintain strong control of the FWER at level 0.05. Tests are designed and conducted with *K* = 4, *ρ* = 0.4, $w_{E}^{2}=w_{S}^{2}=0.5$ and *σ*_*E*_=*σ*_***S***_=1. Results are based on 100000 simulations for each parameter configuration. The dashed horizontal line corresponds to the nominal FWER and the dotted line to the upper bound for simulation error.

Figure 3 provides empirical FWERs for parameter configurations of the from $\theta_{E}=(\theta_{E1},\ldots ,\theta_{\mathrm{EK}}),\;\theta_{S}=(\theta_{S1},\ldots ,\theta_{\mathrm{SK}})$, where one parameter of each pair (*θ*_*Ei*_,*θ*_*Si*_) is large and the other zero to evaluate the conjecture made in Section 2.2, which designs will control these at the nominal level *α*. For the purpose of this evaluation, the large effect was set to 1 million, and 100000‐fold simulations are used. From the graph, it can be seen that the FWER is well controlled for any parameter configuration, as conjectured.

**Figure 4:**
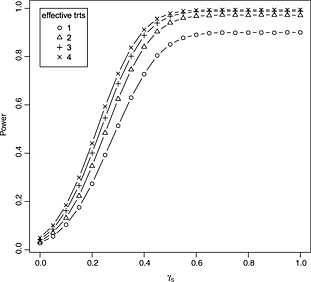
Empirical power to correctly reject at least one false null hypothesis of tests of the form (3) designed to maintain strong control of the FWER at level 0.05 and attain power 0.9 under ***θ***_*E*_=(0.178,0.178,0.178,0.545) (when all treatments are safe). Tests are designed and conducted with *K* = 4, $w_{E}^{2}=w_{S}^{2}=0.5$, *ρ* = 0.4 and *σ*_*E*_=*σ*_***S***_=1. Power is evaluated under configurations of ***θ*** with ***θ***_***S***_=(*γ*_***S***_,…,*γ*_***S***_) and ***θ***_*E*_=(*δ*_0_,…,*δ*_0_,*δ*,…,*δ*) such that *j* treatments have the desired effect for efficacy (*δ* = 0.545) and the remaining have the minimum clinically important effect (*δ*_0_=0.178). Results are based on 100000 simulations for each parameter configuration.

Figure 4 shows how the power of the procedure changes as the safety of the experimental treatments changes. Results are presented for one to four treatments exhibiting the desired effect for efficacy of 0.545, while the remaining have the minimum clinically important effect of 0.178. Power increases as the safety of the treatments increases and reaches the desired level of 0.9 for a safety effect of around 0.5. Power also increases as the number of treatments with the desired efficacy increases, although this increase diminishes somewhat with the number of efficacious treatments.

### 3.3 Misspecification of *ρ*

**Figure 5:**
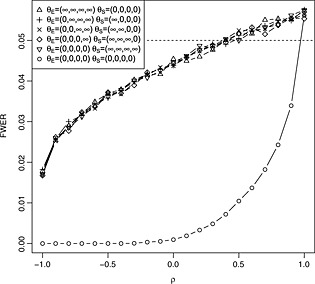
Empirical FWERs of tests of the form (3) designed to maintain strong control of the FWER at level 0.05 and attain power 0.9 under ***θ***_*E*_=(0.178,0.178,0.178,0.545) (when all treatments are safe). Tests are designed and conducted with *K* = 4, $w_{E}^{2}=w_{S}^{2}=0.5$ and *σ*_*E*_=*σ*_***S***_=1. Tests are also designed assuming *ρ* = 0.4 but conducted for a range of correlations. Empirical error probabilities are evaluated under configurations of ***θ*** with ***θ***_***S***_=(*γ*_***S***_,…,*γ*_***S***_) and ***θ***_*E*_=(*γ*_*E*_,…,*γ*_*E*_). Results are based on 100000 simulations for each scenario.

When specifying our model, we have so far assumed that response variances and their correlation are known. In this section, we will investigate the robustness of our design to the assumption of known correlation. Figure 5 shows the simulated FWER based on 100000 simulations of tests as the correlation between endpoints varies. Six different true parameter constellations are considered, namely, the global null hypothesis and five ‘worst‐case’ configurations (once again using 1 million instead of infinity for simulation purposes). For all six settings, the FWER is controlled at the design value *ρ* = 0.4, and the procedure is conservative for all correlations below this. Under the global null hypothesis, only perfect correlation results in an inflation of the FWER, while it is inflated once the true correlation is above the design value for the worst‐case configurations. The maximum inflation, achieved under perfect positive correlation, is, however, small at 10% of the nominal value of the FWER.

Tamhane *et al.*, 26 observe that typically either the correlation is assumed to be known (as performed here) or a correlation of one is treated as the worst‐case scenario. Given the relative conservatism of the proposed procedure at reasonable values of the parameters, we believe the former is sufficient, although the latter would certainly also be possible. A more elegant solution given in 26 overcomes this problem by estimating the correlation mid‐study and uses an approach due to Berger & Boos 27 to obtain an upper bound for the FWER accounting for the sampling error of the sample correlation coefficient.

## 4 Discussion

In this paper, we have presented an approach for designing MAMS studies based on a joint model for efficacy and safety data, which considers both endpoints when selecting the most promising treatment for further investigation and tests the efficacy and safety of the selected treatment relative to control. The main challenge with obtaining the relevant distributions of the test statistics arose from the requirement to select from treatments satisfying a minimum safety requirement. We have shown that the FWER is strongly controlled under the assumption that effects of different treatments for the same endpoint have the same sign. Our simulation results show, however, that strong control of the FWER also appears to hold when this assumption is not made.

In the presentation and derivations, we have made a number of assumptions that may not be appropriate for specific settings. For example, single‐stage designs are formulated assuming patient responses follow a bivariate normal distribution with a common correlation between efficacy and safety responses across the active and control treatments. In addition, calculations assume that at the end of Stage 1, there is a common information level for *θ*_*E*,1_,…,*θ*_*E*,*K*_ and a common information level for *θ*_*S*,1_,…,*θ*_*S*,*K*_. This joint distribution will not in general apply if data do not follow a normal distribution because information levels and correlation coefficients may depend on unknown parameters, such as response rates in the case of binary data (see section 9 of 26). One potential solution would be to approximate and derive test boundaries setting the correlation coefficient and information levels to the values that would apply under ***θ***_*E*_=***θ***_***S***_=**0**. However, further simulations would be needed to verify whether this approach would maintain strong control of the FWER at a level close to the nominal value.

Another simplifying assumption we have made is to propose designs setting the safety threshold to be zero, so that only treatments with better safety than control can be selected. A simple shift of the safety test statistic can be used to allow for different thresholds to be used. Similarly, it may be desirable to select treatments only based on efficacy provided that the treatment is safe enough. Simply setting the weight on safety within the objective function to zero can accomodate this situation. Finally, as outlined before, it may not be appropriate to test for superiority in terms of safety over control. Shifting the respective test statistics for safety will allow non‐inferiority hypothesis to be used instead. It is also easy to envisage application of this design in other settings, such as mental health trials, where there are co‐primary efficacy endpoints. In these cases, no minimal threshold would be applied to either efficacy endpoint – the ideas of this work apply to this, somewhat simpler, situation setting the threshold *c* =− *∞*.

One great benefit of multi‐stage clinical trials is their reduced expected sample size compared with single‐stage designs. Such gains can, however, only be realized, if the primary endpoint is observed quickly relative to the recruitment time 28. When this is not the case, it would be of interest to investigate whether methods such as the one described in 29 can be extended to make selection decisions based on intermediate endpoints in the setting discussed in this paper.

Another area for further work regards how to calculate confidence intervals on termination of tests of the form (3). The procedure based around hypothesis testing described here allows almost formulaic decisions about the superiority of experimental treatments over control. It is essential, however, that confidence intervals should also be available to inform decision makers about the probable sizes of any efficacy and safety benefits, in order to give a complete description of the evidence supporting a selected treatment. A future work will be necessary to evaluate if related work 14, 30 can be utilized to obtain interval estimates as well.

## Supporting information

Supporting info item
