# Supplementary material for: Designing multi‐arm multi‐stage clinical trials using a risk–benefit criterion for treatment selection
Source: Stat Med. 2015 Oct 12;35(4):522–33. doi: 10.1002/sim.6760 (PMC4950037; doi:10.1002/sim.6760)
Supplement: Supplementary file 1 — Supporting info item [file SIM-35-522-s001.zip › 3M-Supplement-15SEP2015-V1.pdf]

Research Article

Statistics in Medicine

Received XXXX

(www.interscience.wiley.com) DOI: 10.1002/sim.0000

Designing multi-arm multi-stage clinical trials using a risk-benefit criterion for treatment selection. Supplementary materials.

Thomas Jaki[a]*, Lisa V. Hampson[a]

A.1 Joint distribution of efficacy and safety score statistics under general configurations of θ

We adopt the following notation to let $f_X(x)$ denote the probability density function (pdf) of a random variable X evaluated at $X = x$ and let $f_{X|Y}(x|y)$ denote the conditional pdf of X given $Y = y$. Recall that $\mathcal{I}_T = n/(2\sigma_T^2)$, for $T \in \{E, S\}$, is the common information level for estimating θ_{Tk} in Stage 1 when n subjects are randomised to each trial arm. For each treatment $k = 0, 1, \ldots, K$, we can define statistics

$$\begin{pmatrix} X_{Ek} \\ X_{Sk} \end{pmatrix} = \begin{pmatrix} \mathcal{I}_E(\hat{\mu}_{Ek} - \mu_{E0}) \\ \mathcal{I}_S(\hat{\mu}_{Sk} - \mu_{S0}) \end{pmatrix} \sim N\left(\begin{pmatrix} \mathcal{I}_E\,\theta_{Ek} \\ \mathcal{I}_S\,\theta_{Sk} \end{pmatrix}, \begin{pmatrix} \mathcal{I}_E/2 & \rho\sqrt{(\mathcal{I}_E\mathcal{I}_S)}/2 \\ \rho\sqrt{(\mathcal{I}_E\mathcal{I}_S)}/2 & \mathcal{I}_S/2 \end{pmatrix} \right),$$

where $\theta_{E0} = \theta_{S0} = 0$ and pairs of statistics for different treatments are independent. Since these statistics are functions of μ_{E0} and μ_{S0} rather than their MLEs, they are not directly observable unless average response rates on control are known. However, taking differences between $X_{E,k}$ and $X_{E,0}$, and $X_{S,k}$ and $X_{S,0}$, gives the efficient score statistics Z_{Ek} and Z_{Sk}, respectively. In the following, we shall find it useful to write the objective functions determining treatment selection in terms of the X_{Tk}s, that is,

$$O_k(X_{E0}, X_{S0}) = \frac{w_E(X_{Ek} - X_{E0})}{\sqrt{\mathcal{I}_E}} + \frac{w_S(X_{Sk} - X_{S0})}{\sqrt{\mathcal{I}_S}} \qquad \text{for } k = 1, \ldots, K,$$

where we adopt this notation to emphasise the dependence of objective functions on X_{E0} and X_{S0}.

[a]*Medical and Pharmaceutical Statistics Research Unit, Department of Mathematics and Statistics, Lancaster University, Lancaster, UK*

**Correspondence to: Thomas Jaki, Medical and Pharmaceutical Statistics Research Unit, Department of Mathematics and Statistics, Lancaster University, Lancaster, UK. E-mail: jaki.thomas@gmail.com*

Copyright © 2015 John Wiley & Sons, Ltd.

Prepared using simauth.cls *[Version: 2010/03/10 v3.00]*

Let $\mathcal{C} = \{X_{Ek} = x_E, X_{Sk} = x_S, X_{E0} = x_{E0}, X_{S0} = x_{S0}\}$. The conditional joint density of $(X_{Ei^\star}, X_{Si^\star}, X_{E0}, X_{S0}, i^\star)$ given $N_S \geq 1$ evaluated at $(x_E, x_S, x_{E0}, x_{S0}, k)$ can be written as

$$\mathbb{P}\{i^\star = k \mid \mathcal{C},\, N_{\mathcal{S}} \geq 1; \boldsymbol{\theta}\}\, f_{X_{Ek},X_{Sk},X_{E0},X_{S0}}(x_E, x_S, x_{E0}, x_{S0} \mid N_{\mathcal{S}} \geq 1; \boldsymbol{\theta}) \qquad \text{if } x_S \geq x_{S0}. \tag{1}$$

Under the proposed treatment selection criterion, the LHS selection probability in Equation (1) is the conditional probability that treatment k satisfies the minimum safety criterion for selection *and* strikes a superior risk-benefit trade-off to all other competing treatments (if any) meeting the same safety requirement. Thus, in our notation, treatment k is selected if and only if $X_{Sk} > X_{S0}$ and for all treatments m, $m \neq k$, either $X_{Sm} \leq X_{S0}$ or $O_m < O_k$. Let $O_k(x_{E0}, x_{S0})$ represent the risk-benefit objective function for treatment k given $X_{E0} = x_{E0}$ and $X_{S0} = x_{S0}$. Then, $\mathbb{P}\{i^\star = k | \mathcal{C}, N_{\mathcal{S}} \geq 1; \boldsymbol{\theta}\}$ can be written as

$$\mathbb{P}\left\{ \bigcap_{m \neq k} \{O_m(x_{E0}, x_{S0}) < q^\star\} \cup \{X_{Sm} \leq x_{S0}\} \,\middle|\, \mathcal{C}, X_{Sk} > X_{S0}, N_{\mathcal{S}} \geq 1; \boldsymbol{\theta} \right\} \mathbb{P}\{X_{Sk} > X_{S0} \mid \mathcal{C}, N_{\mathcal{S}} \geq 1; \boldsymbol{\theta}\}, \tag{2}$$

where $q^\star$, a function of $(x_E,\, x_S,\, x_{E0},\, x_{S0})$, is the value of $O_k(X_{E0}, X_{S0})$ consistent with the conditioning event $\mathcal{C}$. The conditioning events on the LHS of (2) can be simplified by noting that their intersection is precisely $\mathcal{C}$. Furthermore, since pairs $(X_{E0},\, X_{S0}), \ldots, (X_{EK},\, X_{SK})$ are independent, it follows that we can simplify expression (2) so that it becomes

$$\mathbb{P}\left\{ \bigcap_{m \neq k} \{O_m(x_{E0}, x_{S0}) < q^\star\} \cup \{X_{Sm} \leq x_{S0}\}; \boldsymbol{\theta} \right\} \mathbb{P}\{X_{Sk} > X_{S0} \mid \mathcal{C}, N_{\mathcal{S}} \geq 1; \boldsymbol{\theta}\}. \tag{3}$$

For treatment m, $(O_m(x_{E0}, x_{S0}),\, X_{Sm})$ follow a bivariate normal joint distribution with mean $\boldsymbol{\mu}_m$ and variance $\boldsymbol{\Sigma}$ given by

$$\boldsymbol{\mu}_m = \begin{pmatrix} \frac{w_E(\mathcal{I}_E\theta_{Em} - x_{E0})}{\sqrt{\mathcal{I}_E}} + \frac{w_S(\mathcal{I}_S\theta_{Sm} - x_{S0})}{\sqrt{\mathcal{I}_S}} \\ \mathcal{I}_S\theta_{Sm} \end{pmatrix}, \qquad \boldsymbol{\Sigma} = \begin{pmatrix} 0.5 + \rho w_E w_S & \sqrt{\mathcal{I}_S}(w_E\rho + w_S)/2 \\ \sqrt{\mathcal{I}_S}(w_E\rho + w_S)/2 & \mathcal{I}_S/2 \end{pmatrix}. \tag{4}$$

Pairs $(O_{m_1}(x_{E0}, x_{S0}),\, X_{Sm_1})$ and $(O_{m_2}(x_{E0}, x_{S0}),\, X_{Sm_2})$, for $m_1 \neq m_2$, are conditionally independent given $X_{E0} = x_{E0}$ and $X_{S0} = x_{s0}$. Therefore, the probability that all treatments other than treatment k fail to meet either the minimum safety criterion or the risk-benefit cut-off $q^\star$, can be written as the product of $(K - 1)$ bivariate normal probabilities that can be computed using standard software such as the R package `mvtnorm` [1,2].

Substituting expression (3) for $\mathbb{P}\{i^\star = k \mid \mathcal{C}, N_{\mathcal{S}} \geq 1; \boldsymbol{\theta}\}$ in (1) and noting that $\mathbb{P}\{N_{\mathcal{S}} \geq 1 \mid X_{Sk} > X_{S0}; \boldsymbol{\theta}\} = 1$, we can re-arrange (1) using properties of conditional probabilities to find that the conditional joint density of $(X_{Ei^\star}, X_{Si^\star}, X_{E0}, X_{S0}, i^\star)$ given $N_{\mathcal{S}} \geq 1$ is

$$\mathbb{1}\{x_S > x_{S0}\} \frac{f_{X_{Ek},X_{Sk}}(x_E, x_S; \boldsymbol{\theta}) f_{X_{E0},X_{S0}}(x_{E0}, x_{S0})}{\mathbb{P}\{N_{\mathcal{S}} \geq 1; \boldsymbol{\theta}\}} \prod_{m \neq k} \mathbb{P}\{\{O_m(x_{E0}, x_{S0}) < q^\star\} \cup \{X_{Sm} \leq x_{S0}\}; \boldsymbol{\theta}\}, \tag{5}$$

where $\mathbb{1}\{x > y\}$ is the indicator function taking the value 1 if $x > y$ and 0 otherwise. The conditional joint density of $(Z_{Ei^\star}, Z_{Si^\star}, i^\star)$ follows by application of the convolution formula to Equation (5) to obtain:

$$\begin{aligned} & f_{Z_{Ei^\star},Z_{Si^\star},i^\star}(z_E, z_S, k \mid N_{\mathcal{S}} \geq 1; \boldsymbol{\theta}) \\ &= \frac{\mathbb{1}\{z_S > 0\}}{\mathbb{P}\{N_{\mathcal{S}} \geq 1; \boldsymbol{\theta}\}} \int_{-\infty}^{\infty} \int_{-\infty}^{\infty} \prod_{m \neq k} [1 - \mathbb{P}\{\{O_m(x_E - z_E, x_S - z_S) \geq \ell_1(z_E, z_S)\} \cap \{X_{Sm} \geq \ell_2(x_S, z_S)\}; \boldsymbol{\theta}\}] \\ &\qquad \times f_{X_{Ek},X_{Sk}}(x_E, x_S; \boldsymbol{\theta}) f_{X_{E0},X_{S0}}(x_E - z_E, x_S - z_S; \boldsymbol{\theta}) \mathrm{d}x_E\, \mathrm{d}x_S, \end{aligned} \tag{6}$$

Copyright © 2015 John Wiley & Sons, Ltd.

Prepared using simauth.cls

where $\ell_2(x_S, z_S) = x_S - z_S$ and we write $q^\star$ as $\ell_1(z_E, z_S) = (w_E z_E)/\sqrt{\mathcal{I}_E} + (w_S z_S)/\sqrt{\mathcal{I}_S}$ to emphasise the dependence of this threshold on z_E and z_S.

When patients are randomised to each active treatment and control in a common $r:1$ ratio, it is straightforward to generalise the above arguments to derive the joint distribution of $(Z_{Ei^\star}, Z_{Si^\star}, i^\star)$ by setting $\mathcal{I}_T = rn/\{(r+1)\sigma_T^2\}$, for $T \in \{E, S\}$, and modifying accordingly the joint distribution of $(X_{E0},\, X_{S0})$ and for each $k = 1, \ldots, K$, of $(X_{Ek},\, X_{Sk})$ and $(O_k,\, X_{Sk})$.

A.2 Limiting marginal densities of $Z_{Ei^\star}$ and $Z_{Si^\star}$ under configurations of θ maximising the FWER

We use the results of the previous section to derive limiting conditional densities of $Z_{Ei^\star}$ and $Z_{Si^\star}$ given $N_S \geq 1$ under the configurations of $\boldsymbol{\theta}$ identified in Section 2.1 of the main manuscript as corresponding to local maxima of the FWER. These effect configurations are of the general form $\boldsymbol{\theta} = (\boldsymbol{\theta}_E, \boldsymbol{\theta}_S)$ with $\boldsymbol{\theta}_E = (\gamma_E, \ldots, \gamma_E)$ and $\boldsymbol{\theta}_S = (\gamma_S, \ldots, \gamma_S)$. For the purposes of FWER calculations we wish to find

1. the limit of the conditional distribution of $Z_{Ei^\star}$ given $N_S \geq 1$ under $\gamma_E = 0$ and letting $\gamma_S \to \infty$;
2. the limit of the conditional distribution of $Z_{Si^\star}$ given $N_S \geq 1$ under $\gamma_S = 0$ and letting $\gamma_E \to \infty$.

We proceed to derive these limiting distributions in the next sections.

2.1. Limiting marginal conditional distribution of $Z_{Ei^\star}$ given $N_S \geq 1$

Fix $\gamma_E = 0$ and let γ_S take any arbitrary finite value. Under the configuration of $\boldsymbol{\theta}$ thus defined, pairs $(O_{m_1}(x_{E0}, x_{S0}),\, X_{Sm_1})$ and $(O_{m_2}(x_{E0}, x_{S0}),\, X_{Sm_2})$, for $m_1 \neq m_2$, are independent *and* identically distributed. Therefore, for $m = 1, \ldots, K$,

$$\begin{aligned}\mathbb{P}\{\{O_m(x_E - z_E, x_S - z_S) \geq \ell_1(z_E, z_S)\} \cap \{X_{Sm} \geq \ell_2(x_S, z_S)\}\,; \gamma_E = 0, \gamma_S\} \\ = \int_{\ell_2(x_S,z_S)-\mathcal{I}_S\gamma_S}^{\infty-\mathcal{I}_S\gamma_S} \int_{\ell_1(z_E,z_S)}^{\infty} f_{X_{S0}}(u) f_{O_1(x_E-z_E,x_S-z_S)|X_{S1}}(t \mid u + \mathcal{I}_S\gamma_S; \gamma_E = 0, \gamma_S) \mathrm{d}t\, \mathrm{d}u, \end{aligned} \tag{7}$$

with $\ell_1(z_E, z_S)$ and $\ell_2(x_S, z_S)$ as defined in Appendix A.1. The integrand in Equation (7) is a product of pdfs and so, by Fubini's Theorem, we are free to choose the order of integration. The conditional distribution of $O_1(x_E - z_E, x_S - z_S)$ given $X_{S1} = u + \mathcal{I}_S\gamma_S$ can be deduced from the joint distribution of these random variables defined by Equation (4).

Integrating out $Z_{Si^\star}$ from the joint conditional density of $(Z_{Ei^\star},\, Z_{Si^\star},\, i^\star)$ and transforming variables of integration by setting $y = x_S - \mathcal{I}_S\gamma_S$ and $\psi = z_S - \mathcal{I}_S\gamma_S$, we obtain:

$$\begin{aligned} f_{Z_{Ei^\star}, i^\star}(z_E, k \mid N_S \geq 1; \gamma_E = 0, \gamma_S) = \frac{1}{\mathbb{P}\{N_S \geq 1; \gamma_S\}} \int_{-\mathcal{I}_S\gamma_S}^{\infty-\mathcal{I}_S\gamma_S} \int_{-\infty}^{\infty} \int_{-\infty-\mathcal{I}_S\gamma_S}^{\infty-\mathcal{I}_S\gamma_S} f_{X_{E0},X_{S0}}(x_E, y) f_{X_{E0},X_{S0}}(x_E - z_E, y - \psi) \\ \times A(\gamma_S, x_E, y, \psi)^{K-1} \mathrm{d}y\, \mathrm{d}x_E\, \mathrm{d}\psi, \end{aligned}$$

where, after re-arranging (7) we have

$$A(\gamma_S, x_E, y, \psi) = 1 - \int_{y-\psi-\mathcal{I}_S\gamma_S}^{\infty-\mathcal{I}_S\gamma_S} \int_{\ell_3(x_E,y)}^{\infty} f_{V,U}(v, u) \mathrm{d}v\, \mathrm{d}u$$

Copyright © 2015 John Wiley & Sons, Ltd.

with $\ell_3(x_E,\, y) = (w_E x_E)/\sqrt{\mathcal{I}_E} + (w_S y)/\sqrt{\mathcal{I}_S}$ and marginally $V \sim N(0, 0.5 + w_E w_S \rho)$, $U \sim N(0, \mathcal{I}_S/2)$ and $\text{Cov}(V, U) = (w_E\rho + w_S)\sqrt{\mathcal{I}_S}/2$.

Given a pair of boundaries for monitoring $(Z_{Ei^\star}, Z_{Si^\star})$, the probability of making a familywise error at the test's first stage is

$$\mathbb{P}\{Z_{Ei^\star} \geq u_E, N_{\mathcal{S}} \geq 1; \gamma_E = 0, \gamma_S\} = K \int_{-\mathcal{I}_S\gamma_S}^{\infty-\mathcal{I}_S\gamma_S} \int_{-\infty}^{\infty} \int_{-\infty-\mathcal{I}_S\gamma_S}^{\infty-\mathcal{I}_S\gamma_S} f_{X_{E0},X_{S0}}(x_E, y) f_{X_{S0}}(y-\psi) A(\gamma_S, x_E, y, \psi)^{K-1} \times \Phi\left(\frac{x_E - \sqrt{(\mathcal{I}_E/\mathcal{I}_S)}\rho(y-\psi) - u_E}{\sqrt{0.5\,\mathcal{I}_E\,(1-\rho^2)}}\right) \mathrm{d}y\, \mathrm{d}x_E\, \mathrm{d}\psi. \quad (8)$$

We wish to find the limit of this probability as $\gamma_S \to \infty$ in Equation (8). This is not straightforward because both the integrand and limits of the multivariate integral in (8) are functions of γ_S. The dependence of integral limits on γ_S can be removed by multiplying the integrand by $\mathbb{1}\{-\infty - \mathcal{I}_S\gamma_S < y < \infty - \mathcal{I}_S\gamma_S\}$ and $\mathbb{1}\{-\mathcal{I}_S\gamma_S < \psi < \infty - \mathcal{I}_S\gamma_S\}$. To consider the limiting behaviour of this new integrand as $\gamma_S \to \infty$, let $\{\gamma_{S,\tau}\}_{\tau=1}^{\infty}$ be an unbounded sequence of increasing values of γ_S. Setting

$$\begin{aligned} h_\tau(x_E, y, \psi) =& f_{X_{E0},X_{S0}}(x_E, y) f_{X_{S0}}(y-\psi) \Phi\left(\frac{x_E - \sqrt{(\mathcal{I}_E/\mathcal{I}_S)}\rho(y-\psi) - u_E}{\sqrt{0.5\,\mathcal{I}_E\,(1-\rho^2)}}\right) \\ &\times [1 - \mathbb{P}\{y - \psi - \mathcal{I}_S\gamma_{S,\tau} < U < \infty - \mathcal{I}_S\gamma_{S,\tau}, V \geq \ell_3(x_E, y)\}]^{K-1} \\ &\times \mathbb{1}\{-\infty - \mathcal{I}_S\gamma_{S,\tau} < y < \infty - \mathcal{I}_S\gamma_{S,\tau}\} \mathbb{1}\{-\mathcal{I}_S\gamma_{S,\tau} < \psi < \infty - \mathcal{I}_S\gamma_{S,\tau}\}, \end{aligned}$$

this sequence of non-negative measurable functions is bounded above by the integrable function $g(\psi, x_E, y) = 2^{K-1} f_{X_{E0},X_{S0}}(x_E, y) f_{X_{S0}}(y-\psi)$. As $\tau \to \infty$, applying Lebesgue's Monotone Convergence Theorem to find the limit of $\mathbb{P}\{y - \psi - \mathcal{I}_S\gamma_{S,\tau} < U < \infty - \mathcal{I}_S\gamma_{S,\tau}, V \geq \ell_3(x_E, y)\}$, we obtain

$$h_\tau(x_E, y, \psi) \to f_{X_{E0},X_{S0}}(x_E, y) f_{X_{S0}}(y-\psi) \mathbb{P}\{V \leq \ell_3(x_E, y)\}^{K-1} \Phi\left(\frac{x_E - \sqrt{(\mathcal{I}_E/\mathcal{I}_S)}\rho(y-\psi) - u_E}{\sqrt{0.5\,\mathcal{I}_E\,(1-\rho^2)}}\right).$$

By Lebesgue's Dominated Convergence Theorem, we may interchange integration and limits in Equation (8) to write

$$\lim_{\gamma_S \to \infty} \mathbb{P}\{Z_{Ei^\star} \geq u_E, N_{\mathcal{S}} \geq 1; \gamma_E = 0, \gamma_S\} = K \int_{-\infty}^{\infty} \int_{-\infty}^{\infty} \int_{-\infty}^{\infty} f_{X_{E0},X_{S0}}(x_E, y) f_{X_{S0}}(y-\psi) \Phi\left(\frac{\ell_3(x_E, y)}{\sqrt{(0.5 + w_E w_S \rho)}}\right)^{K-1} \times \Phi\left(\frac{x_E - \sqrt{(\mathcal{I}_E/\mathcal{I}_S)}\rho(y-\psi) - u_E}{\sqrt{0.5\,\mathcal{I}_E\,(1-\rho^2)}}\right) \mathrm{d}y\, \mathrm{d}x_E\, \mathrm{d}\psi.$$

2.2. *Limiting marginal conditional distribution of $Z_{Si^\star}$ given $N_{\mathcal{S}} \geq 1$*

Following a similar approach to that taken in Appendix A.2.1, we proceed by integrating out $Z_{Ei^\star}$ from $f_{Z_{Ei^\star},Z_{Si^\star},i^\star}(z_E, z_S, k \mid N_{\mathcal{S}} \geq 1; \boldsymbol{\theta})$ to obtain

$$\begin{aligned} f_{Z_{Si^\star},i^\star}(z_S, k | N_{\mathcal{S}} \geq 1; \gamma_E, \gamma_S = 0) =& \frac{\mathbb{1}\{z_S > 0\}}{\mathbb{P}\{N_{\mathcal{S}} \geq 1; \gamma_S = 0\}} \\ &\times \int_{-\infty-\mathcal{I}_E\gamma_E}^{\infty-\mathcal{I}_E\gamma_E} \int_{-\infty-\mathcal{I}_E\gamma_E}^{\infty-\mathcal{I}_E\gamma_E} \int_{-\infty}^{\infty} f_{X_{E0},X_{S0}}(v, x_S) f_{X_{E0},X_{S0}}(v-y, x_S - z_S) \\ &\times [1 - \mathbb{P}\{U \geq x_S - z_S, V \geq \ell_3(\nu, x_S)\}]^{K-1}\, \mathrm{d}x_S\, \mathrm{d}v\, \mathrm{d}y. \end{aligned} \quad (9)$$

 Copyright © 2015 John Wiley & Sons, Ltd.

Only the limits of integration in Equation (9) depend upon γ_E; letting $\gamma_E \to \infty$, it follows from Lebesgue's Monotone Convergence Theorem that these limits can be replaced by $\pm\infty$. The limit of the probability of making a familywise error under the configuration of $\boldsymbol{\theta}$ with $\gamma_S = 0$ and letting $\gamma_E \to \infty$, can be evaluated numerically as:

$$\lim_{\gamma_E \to \infty} \mathbb{P}\{Z_{Si^\star} \geq u_S, N_{\mathcal{S}} \geq 1; \gamma_E, \gamma_S = 0\}$$
$$= K \int_{\max\{0,u_S\}}^{\infty} \int_{-\infty}^{\infty} \int_{-\infty}^{\infty} \int_{-\infty}^{\infty} f_{X_{E0},X_{S0}}(\nu, x_S) f_{X_{E0},X_{S0}}(\nu - y, x_S - z_S) \times$$
$$[1 - \mathbb{P}\{U \geq x_S - z_S, V \geq \ell_3(\nu, x_S)\}]^{K-1} \, \mathrm{d}x_S \, \mathrm{d}\nu \, \mathrm{d}y \, \mathrm{d}z_S.$$

References

1. Genz A, Bretz F, Miwa T, Mi X, Leisch F, Scheipl F, Hothorn T. *mvtnorm: Multivariate Normal and t Distributions* 2010. URL http://CRAN.R-project.org/package=mvtnorm, R package version 0.9-92.
2. Genz A, Bretz F. *Computation of Multivariate Normal and t Probabilities*. Lecture Notes in Statistics, Springer-Verlag: Heidelberg, 2009.

Copyright © 2015 John Wiley & Sons, Ltd.
